# AMPK induces regulatory innate lymphoid cells after traumatic brain injury

**DOI:** 10.1172/jci.insight.126766

**Published:** 2021-01-11

**Authors:** Babak Baban, Molly Braun, Hesam Khodadadi, Ayobami Ward, Katelyn Alverson, Aneeq Malik, Khoi Nguyen, Skon Nazarian, David C. Hess, Scott Forseen, Alexander F. Post, Fernando L. Vale, John R. Vender, Md. Nasrul Hoda, Omid Akbari, Kumar Vaibhav, Krishnan M. Dhandapani

**Affiliations:** 1 Department of Oral Biology and Diagnostic Sciences, Dental College of Georgia, Augusta University, Augusta, Georgia, USA.; 2 Department of Surgery,; 3 Department of Neurology,; 4 Department of Neurosurgery, and; 5 Department of Radiology and Imaging, Medical College of Georgia, Augusta University, Augusta, Georgia, USA.; 6 Department of Neurobiology, Barrow Neurological Institute, Phoenix, Arizona, USA.; 7 Department of Molecular Microbiology and Immunology, Keck School of Medicine, University of Southern California, Los Angeles, California, USA.

**Keywords:** Immunology, Neuroscience, Neurological disorders

## Abstract

The CNS is regarded as an immunoprivileged organ, evading routine immune surveillance; however, the coordinated development of immune responses profoundly influences outcomes after brain injury. Innate lymphoid cells (ILCs) are cytokine-producing cells that are critical for the initiation, modulation, and resolution of inflammation, but the functional relevance and mechanistic regulation of ILCs are unexplored after acute brain injury. We demonstrate increased proliferation of all ILC subtypes within the meninges for up to 1 year after experimental traumatic brain injury (TBI) while ILCs were present within resected dura and elevated within cerebrospinal fluid (CSF) of moderate-to-severe TBI patients. In line with energetic derangements after TBI, inhibition of the metabolic regulator, AMPK, increased meningeal ILC expansion, whereas AMPK activation suppressed proinflammatory ILC1/ILC3 and increased the frequency of IL-10–expressing ILC2 after TBI. Moreover, intracisternal administration of IL-33 activated AMPK, expanded ILC2, and suppressed ILC1 and ILC3 within the meninges of WT and Rag1^–/–^ mice, but not Rag1^–/–^ IL2rg^–/–^ mice. Taken together, we identify AMPK as a brake on the expansion of proinflammatory, CNS-resident ILCs after brain injury. These findings establish a mechanistic framework whereby immunometabolic modulation of ILCs may direct the specificity, timing, and magnitude of cerebral immunity.

## Introduction

Traumatic brain injury (TBI) is a significant worldwide public health issue, killing or debilitating over 3 million people annually (1). The blood-brain barrier (BBB) restricts the entry of peripheral immune cells into the uninjured CNS; however, immune responses develop in a context-specific, spatially and temporally regulated manner after acute brain injuries (2, 3). This pattern is suggestive of a highly regulated process rather than passive entry of immune cells through a disrupted BBB. As the infiltration of peripheral immune cells into the CNS is frequently associated with worse TBI outcomes, defining the mechanisms underlying the immune response to brain injury may identify novel avenues for therapeutic development.

Innate lymphoid cells (ILCs) are functionally diverse immunomodulatory cells that develop from common lymphoid progenitors (1, 4). ILCs lack antigen-specific B cell or T cell receptors, are comparatively less abundant than classical adaptive lymphocytes, and are defined by the absence of known lineage markers (1, 5, 6). Functionally, ILCs release effector cytokines to optimally orchestrate immune responses to distinct challenges (1, 5, 6). Along these lines, ILC1, which induces type I immunity, requires the transcription factor T-bet for lineage commitment and includes both NK cells and IFN-γ–producing ILCs (7). ILC2, which stimulates type II immune responses, are defined by the expression of transcription factors ROR-α and GATA-3 and by production of the cytokines IL-5, IL-9, and IL-13 following activation by IL-25 and IL-33 (8, 9). Finally, ILC3, which mediates type III immune responses, requires the transcription factor RORγt and produces the cytokines IL-17 and IL-22 in response to stimulation with IL-1β and IL-23 (10, 11). Despite the emergent role for ILCs as master immune regulators, the presence and functional importance of ILCs after neurological injury remains poorly defined.

ILCs are enriched at barrier surfaces and mucosal borders to provide a first line of host defense (12, 13). The meninges, which function as a structural barrier to support and protect the CNS, are composed of a thick outer dura mater, a thin fluid impermeable arachnoid mater, and a vascularized pia mater that is directly anchored to the brain. While the CNS is traditionally regarded as an immune privileged organ, specialized lymphatic vessels within the meninges receive chemical signals from within the brain and drain cerebrospinal fluid (CSF) into the deep cervical lymph nodes, providing a conduit to facilitate bidirectional communication between the injured CNS and the peripheral immune system (14, 15). Thus, defining the regulation and functional roles of ILCs within the CNS may inform the development of innovative therapeutic opportunities to harness the beneficial aspects of immune activation, while minimizing the deleterious consequences.

Acute reductions in cerebral blood flow produce regional alterations in brain metabolism, including persistent changes in metabolic rates and energy utilization, and poor clinical outcomes after TBI (16, 17). Moreover, cerebral metabolic perturbations increase neuroinflammation and cognitive decline (18, 19). Interestingly, hemodynamic changes in pial arterioles correlated with changes in cerebral blood flow in a porcine model of TBI, while adherence of infiltrating leukocytes within pial venules temporally correlated with necrotic injury immediately following TBI in rodents (20, 21). This association between metabolic changes within the meninges and subsequent immune activation remains mechanistically undefined; however, immunometabolic changes, including increased glucose uptake, elevated glycolysis, and enhanced activation of the pentose phosphate pathway, are implicated in context-dependent inflammatory activation, while metabolic reprogramming is emerging as a powerful modulator of innate immune regulation (22).

AMPK, an energy sensing serine/threonine kinase that functions as a master metabolic switch, is a heterotrimeric protein complex consisting of a catalytic α and noncatalytic β and -γ subunits. Consistent with the notion that AMPK activation induces a pseudostarvation state to drive antiinflammatory signaling (22), global genetic deletion of AMPKα1 increased proinflammatory activation and induced severe demyelination in a preclinical model of multiple sclerosis (23). In addition, we reported that AMPKα1 was obligatory for antiinflammatory immune polarization and neurological recovery after intracerebral hemorrhage (24); however, the regulatory role for immunometabolic regulation in dynamic ILC polarization remains unexplored. In this study, we used an experimental model of TBI in conjunction with dura and CSF obtained from neurotrauma patients to test the hypothesis that AMPK directs meningeal ILCs after acute brain injury.

## Results

### Elevated presence of ILCs within human meninges and CSF following TBI.

**Figure 1:**
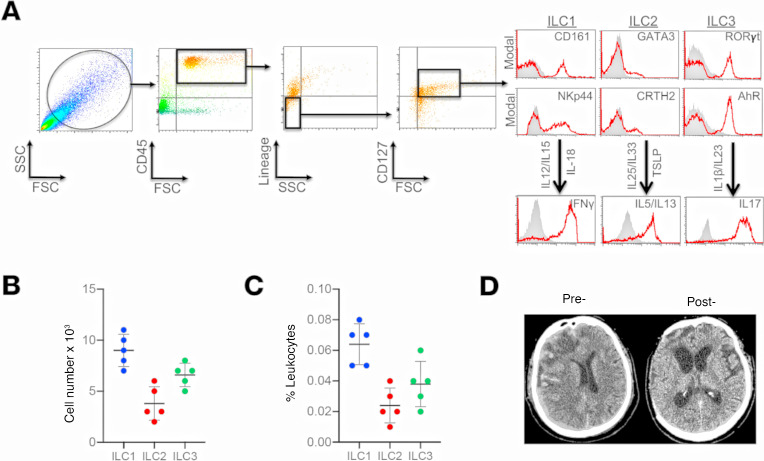
Presence and frequency of ILC subtypes within the meninges of severe TBI patients. (**A**) Dura was collected from consecutive, severe TBI patients undergoing decompressive craniectomy to alleviate elevated intracranial pressure. ILCs were sorted using forward scatter (FSC)/side scatter (SSC) and identified as CD45^+^, lineage-negative (Lin^−^), CD127^+^ lymphoid cells. ILCs subtypes were further defined as ILC1:, CD45^+^Lin^−^CD127^+^CD161^+^NKp44^+^; ILC2, CD45^+^Lin^−^CD127^+^GATA3^+^CRTH2^+^; and ILC3, CD45^+^Lin^−^CD127^+^RORγt^+^AhR^+^, as shown in representative flow cytometry scatterplots. Gray shaded areas indicate isotype controls. To demonstrate functionality, ILCs were further stimulated with cytokine cocktails, and production of signature cytokines was assessed (ILC1, IFN-γ; ILC2, IL-5/IL-13; ILC3, IL-17). (**B** and **C**) Frequency of ILC subtypes from individual patients, expressed as total cell number (**B**) and % leukocytes (**C**) (*n* = 5). Scatterplots depict mean ± SD. (**D**) Computed tomography scan of a TBI patient before (Pre-) and after (Post-) decompressive craniectomy surgery. The dura was collected during surgery at the time of bone flap removal.

**Figure 2:**
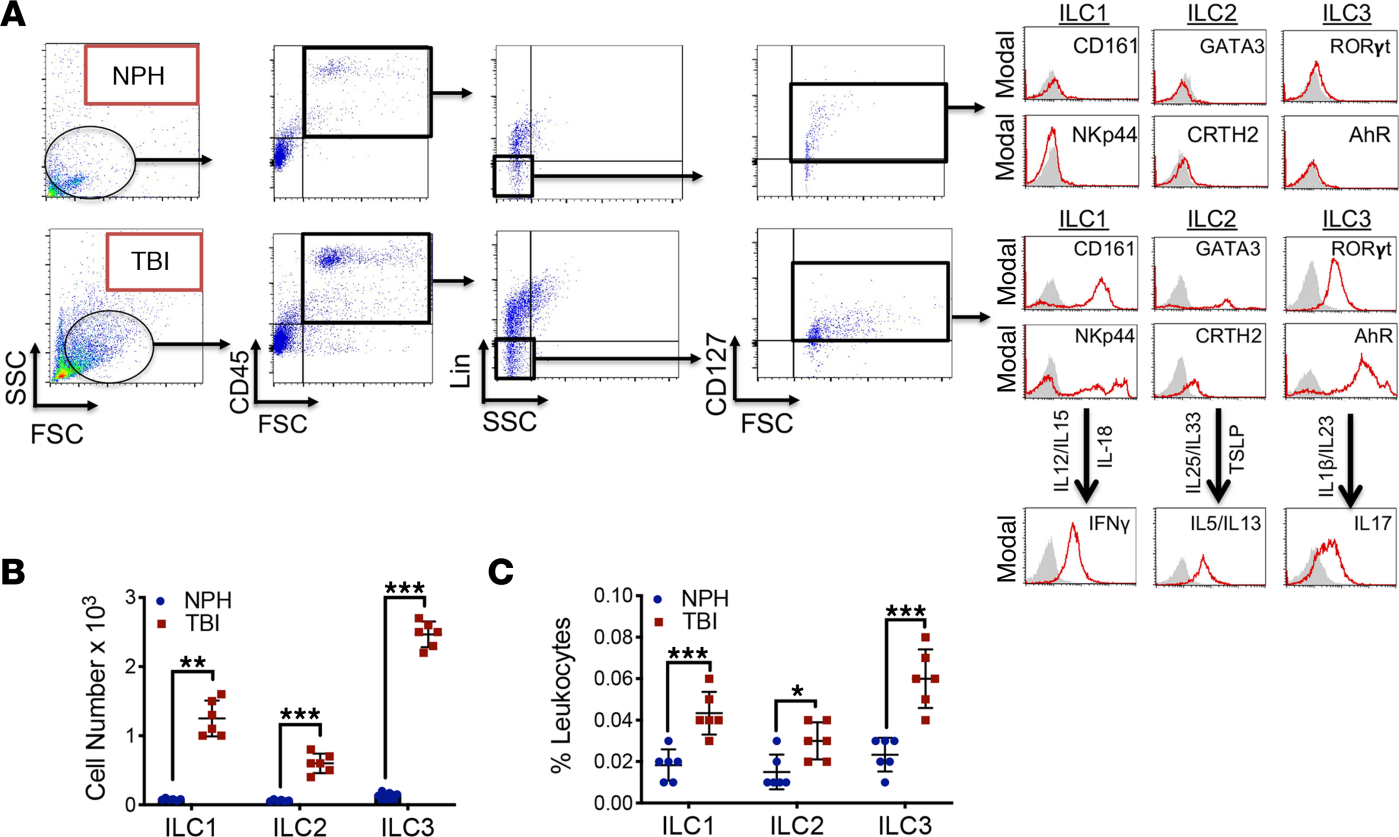
Increased presence of ILC1 and ILC3 within human CSF after TBI. (**A**) CSF was collected from consecutive, adult nontraumatic control (normal pressure hydrocephalus; NPH) or severe TBI patients. Human ILCs were sorted using forward scatter (FSC)/side scatter (SSC) and identified as CD45^+^, lineage-negative (Lin^−^), CD127^+^ lymphoid cells. Selected populations were analyzed for ILC subtype as follows: ILC1, Lin^−^CD127^+^CD161^+^NKp44^+^; ILC2, Lin^−^CD127^+^GATA3^+^CRTH2^+^; and ILC3, Lin^−^CD127^+^AhR^+^RORγt^+^. ILC functionality was further assessed by cytokine production (ILC1, IFN-γ; ILC2, IL-5/IL-13; and ILC3, IL-17) after cytokine stimulation, as shown. Gray shaded areas indicate isotype controls. (**B** and **C**) Quantified data reveal low basal expression of ILC subtypes, with large increases in all ILC classes after TBI. Scatterplots, which are expressed as mean ± SD, depict ILC subtypes as total cell number (**B**) and % leukocytes (**C**). Data from individual patients (*n* = 6 NPH patients, *n* = 6 severe TBI patients) were compared within each ILC subtype using a 2-tailed Student’s *t* test (*P < 0.05, **P < 0.01, ***P < 0.001 versus sham).

The presence of ILCs were initially assessed in dura collected from consecutive TBI patients undergoing a decompressive craniectomy (Figure 1, A–D). Subtype-specific analysis of CD45^+^Lin^–^CD127^+^ ILCs revealed the highest frequencies of CD161^+^NKp44^+^IFN-γ^+^ ILC1 and RORγt^+^AhR^+^IL-17^+^ ILC3, with relatively fewer GATA3^+^CRTH2^+^IL-5^+^IL-13^+^ ILC2 (Figure 1, B and C). We next determined whether ILCs were present within CSF collected from severe TBI patients. Few ILCs were observed within control CSF collected from normal pressure hydrocephalus (NPH) patients (Figure 2, A–C). In contrast, significant elevations in ILC1 (*P* < 0.01 versus NPH), ILC2 (*P* < 0.001 versus NPH), and ILC3 (*P* < 0.001 versus NPH) were observed within CSF derived from TBI patients (Figure 2, B and C).

### ILCs are enriched within murine meninges after experimental TBI.

**Figure 3:**
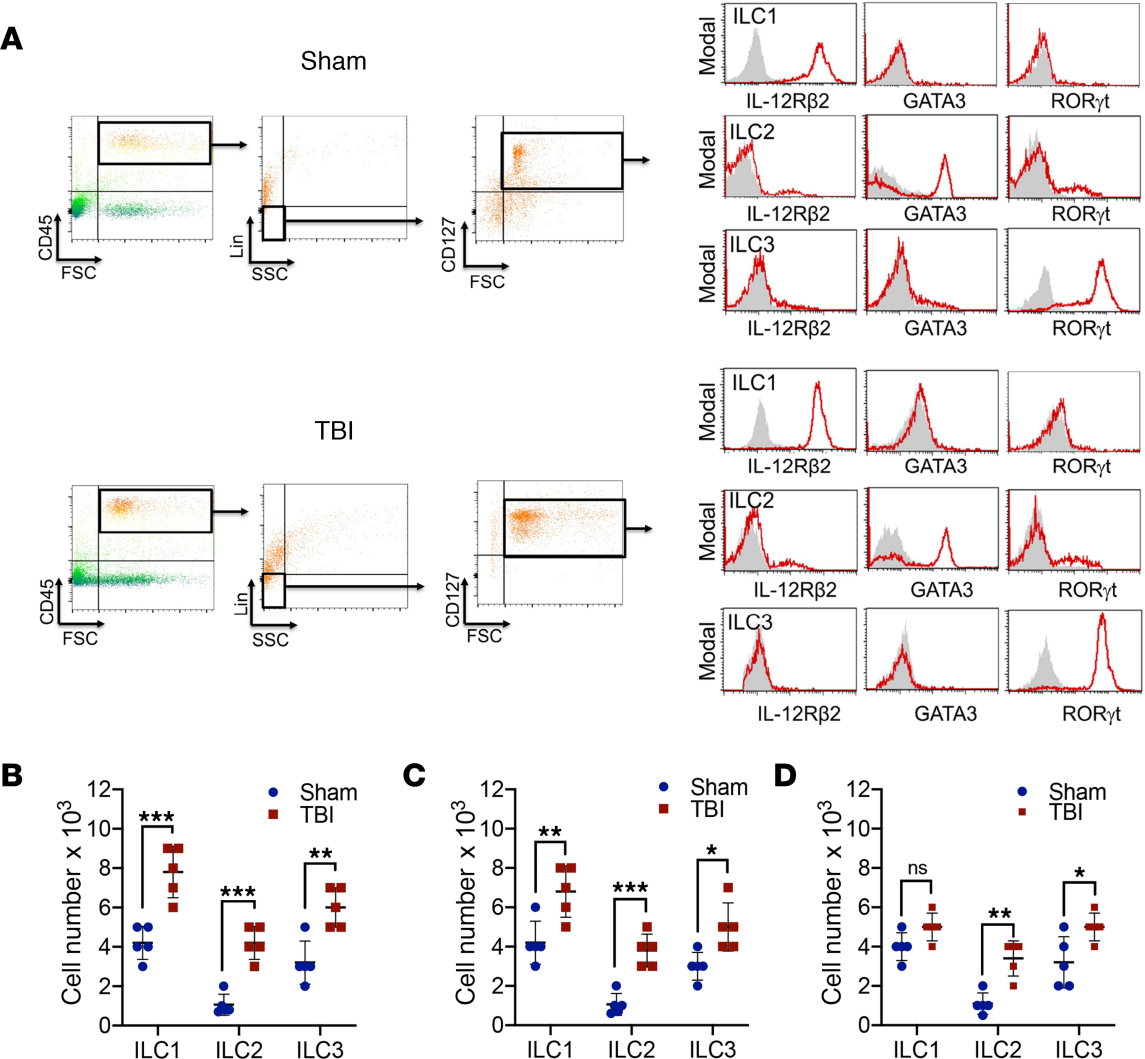
Increased presence of CNS-resident ILCs within the meninges after experimental TBI. (**A**) Representative panels depict multiparameter flow cytometry and gating strategy to identify ILCs in meninges at 1 week after sham or TBI in mixed-sex C57Bl/6J mice. ILCs were sorted using forward scatter (FSC)/side scatter (SSC) and identified as CD45^+^, lineage-negative (Lin^−^), CD127^+^ lymphoid cells. ILC subtypes were further defined as the following: ILC1, CD45^+^Lin^−^CD127^+^IL-12Rβ2^+^; ILC2, CD45^+^Lin^−^CD127^+^GATA3^+^; and ILC3, CD45^+^Lin^−^CD127^+^RORγt^+^. Gray shaded areas indicate isotype controls. (**B**–**D**) Quantified data showing ILCs subtypes at 1 day (**B**), 7 days (**C**), or 1 year (**D**) after TBI. Quantified data (*n* = 5 mice/group) are presented as mean ± SD and compared within each ILC subtype using a Student’s *t* test (*P < 0.05, **P < 0.01, ***P < 0.001 versus sham).

We next ascertained whether changes in ILC incidence similarly occurred following experimental TBI (Figure 3, A–D). Flow cytometric analysis of the murine meninges showed significant increases in ILC1 (*P* < 0.001 versus sham), ILC2 (*P* < 0.001 versus sham), and ILC3 (*P* < 0.01 versus sham) beginning at day 1 after TBI (Figure 3B). Similar increases in all ILC subtypes were present at day 7 after TBI, with elevations in ILC1 (*P* < 0.01 versus sham), ILC2 (*P* < 0.001 versus sham), and ILC3 (*P* < 0.05 versus sham) noted (Figure 3C and Supplemental Figure 2; supplemental material available online with this article; https://doi.org/10.1172/jci.insight.126766DS1). A sustained elevation in ILC2 (*P* < 0.01 versus sham) and ILC3 (*P* < 0.05 versus sham) was maintained at 1 year after TBI (Figure 3D), temporally paralleling the development and persistence of chronic neuroinflammation. In contrast to changes in the meninges, ILCs were not detectable within the brain parenchyma of either sham or TBI mice (data not shown).

### Immunometabolic regulation of ILCs after TBI.

**Figure 4:**
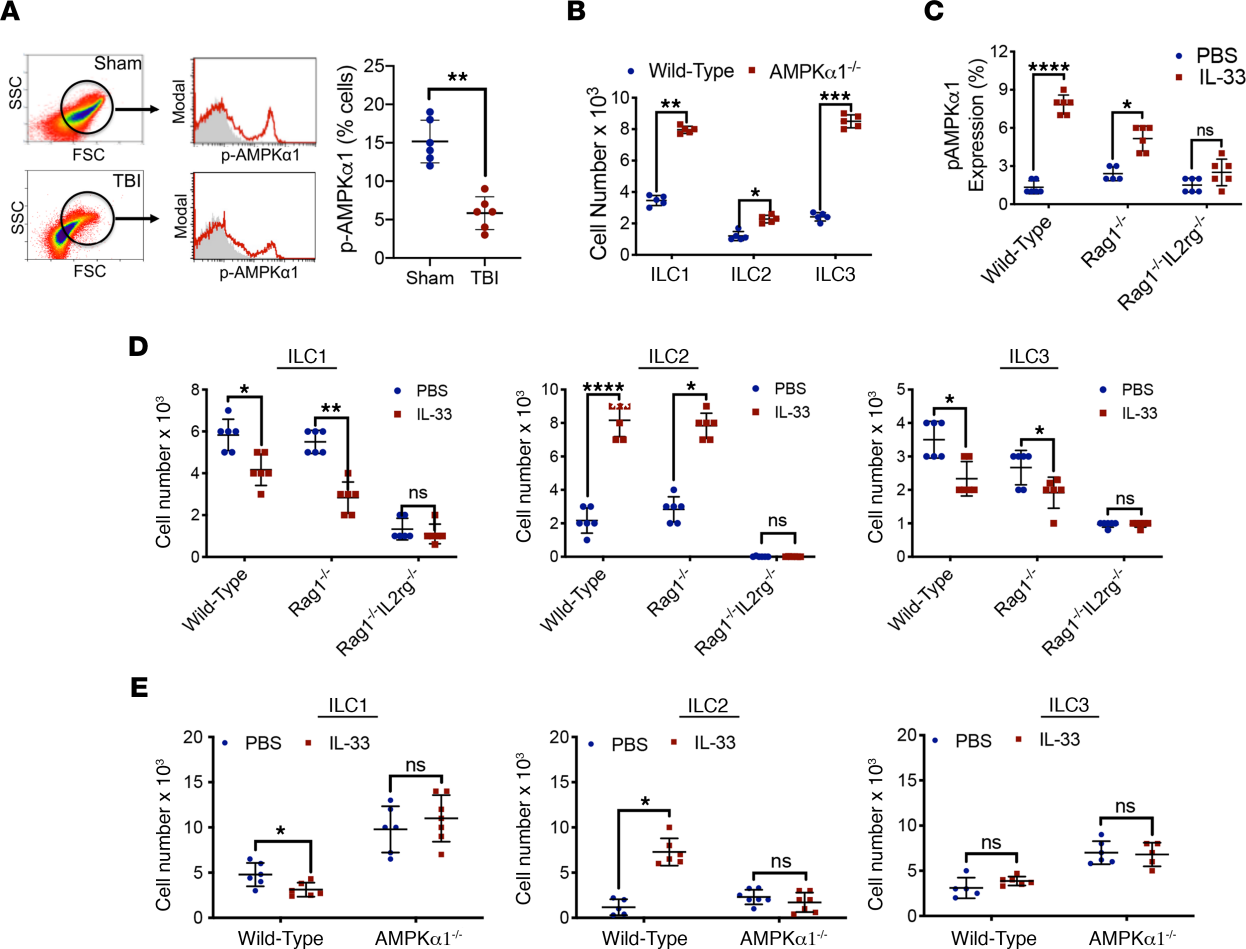
Immunometabolic regulation of ILCs after TBI. (**A**) Phosphorylated AMPKα1 (p-AMPKα1), a measure of AMPK activation, was assessed in meningeal cells at 24 hours after sham/TBI in WT mice. Isolated meninges were assessed by forward scatter (FSC)/side scatter (SSC), and selected populations were further analyzed for p-AMPKα. Scatterplots depicting the % total p-AMPKα^+^ cells are indicative of suppressed AMPK activation within the meninges after TBI. (**B**) AMPKα1–global KO (AMPKα1^–/–^) mice showed higher frequencies of all ILC subtypes after TBI, as compared with WT mice, with most pronounced increases noted for ILC1 and ILC3. (**C**) Intracisternal administration of IL-33 (1 μg) increased meningeal expression of p-AMPKα after TBI, as compared with placebo treatment in both WT mice and in Rag1^–/–^ mice, which lack mature B and T lymphocytes, but possess functional ILC. Conversely, p-AMPKα was unaffected by IL-33 treatment in Rag1^–/–^ IL2rg^–/–^ mice, which lack both mature lymphocytes and ILC2. (**D**) Intracisternal administration of IL-33 (1 μg) increased meningeal expression of ILC2 and suppressed both ILC1 and ILC3 expansion at day 5 after TBI in WT and Rag1^–/–^ mice, as compared with placebo (PBS). In contrast, IL-33 did not affect ILC number in Rag1^–/–^ IL2rg^–/–^ mice, which lack ILC2. Meningeal tissue was analyzed by flow cytometry. (**E**) The stimulatory effects of intracisternal IL-33 on ILC2 frequency were lost in AMPKα1^–/–^ mice, as compared with WT mice. For all panels, quantified data are presented as the mean ± SD from *n* = 6 mice/group. For each panel, data were compared within each ILC subtype using a 2-tailed Student’s *t* test (*P < 0.05, **P < 0.01, ****P < 0.0001).

We next sought to define the mechanisms underlying the differential regulation of ILCs after TBI. Metabolic dysregulation was observed within the meninges, as evidenced by a > 50% reduction in the activation of AMPKα1 after TBI (*P* < 0.01 versus sham) (Figure 4A). The functional importance of this observation was shown by analysis of meningeal ILCs in mice with global KO of AMPKα1 (AMPKα1^–/–^), which revealed an increase in the expansion of all ILC subtypes, with most prominent increases noted for ILC1 (*P* < 0.01 versus sham) and ILC3 (*P* < 0.001 versus sham), as compared with WT mice (Figure 4B). To further dissect the importance of immunometabolic changes in the regulation of ILCs, we performed intracisternal administration of IL-33, an injury-inducible cytokine that promotes ILC2 activation and effector function in mice at 2 hours after TBI. IL-33 increased AMPKα1 phosphorylation within the meninges of both WT (*P* < 0.0001 versus placebo) and Rag1^–/–^ (*P* < 0.05 versus placebo) mice, which lack mature B lymphocytes and T lymphocytes but possess functional ILCs (Figure 4C). In contrast, the stimulatory effects of intracisternal IL-33 on AMPKα phosphorylation were completely lost in Rag1^–/–^ IL2rg^–/–^ mice (Figure 4C), which lack both mature lymphocytes and ILC2. In keeping with the aforementioned data, IL-33 selectively increased ILC2 expansion (*P* < 0.0001 versus placebo in WT, *P* < 0.05 in Rag1^–/–^) while producing a concomitant suppression of ILC1 (*P* < 0.05 versus placebo in WT, *P* < 0.01 in Rag1^–/–^) and ILC3 (*P* < 0.05 versus placebo in WT, *P* < 0.05 in Rag1^–/–^) in both WT and Rag1^–/–^ mice after TBI, while these effects were eliminated in Rag1^–/–^ IL2rg^–/–^ mice (Figure 4D). Moreover, the regulatory effects of IL-33 on ILC cell number were lost in AMPKα1^–/–^ mice, as compared with WT mice (Figure 4E).

### Increased ILC2 is associated with improved neurological outcomes.

**Figure 5:**
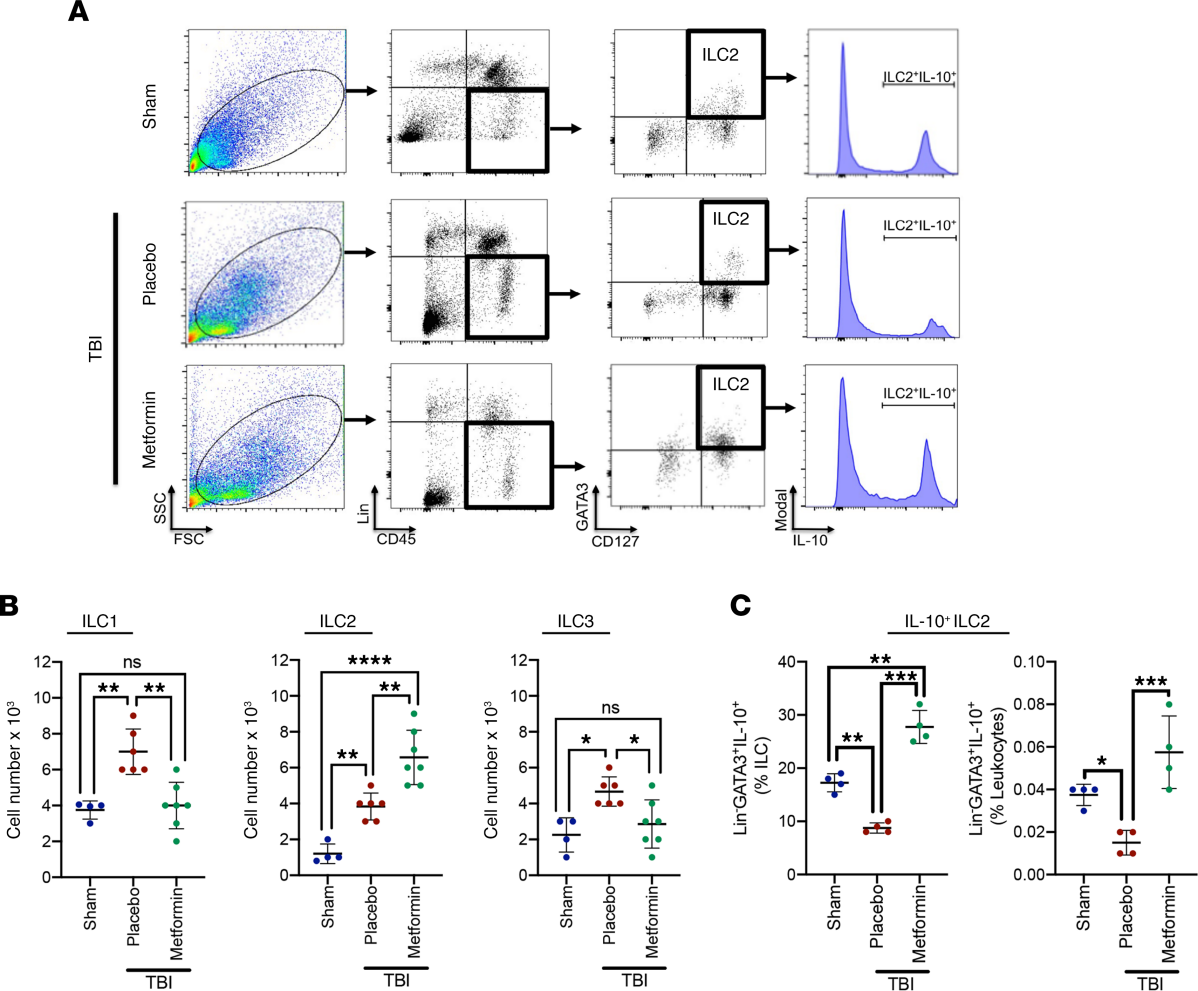
Metformin increased the frequency of ILC2_reg_ after TBI. (**A**) Placebo or Metformin (3 μg) was intracisternally administered at 2 hours after TBI, and isolated meninges were analyzed at day 5 after sham/TBI by forward scatter (FSC)/side scatter (SSC). Lin^–^, CD127^+^, GATA3^+^ ILC2s were gated and further analyzed for the expression of the regulatory cytokine IL-10. Representative panels are provided for each group. (**B** and **C**) Quantification of ILC subtypes (**B**), including IL-10^+^ ILC2_reg_ (**C**). Data are mean ± SD (*n* = 4-7 mice/group). Data were compared using a One-Way ANOVA followed by Tukey’s post-hoc test (*P < 0.05, **P < 0.01, ***P < 0.001, ****P < 0.0001).

To extend the translational relevance of our studies, we next tested whether posttreatment with metformin, an AMPK activator, could regulate CNS-resident ILC expansion. Metformin increased AMPKα1 phosphorylation (*P* < 0.0001 versus placebo treatment; *P* < 0.0001 versus sham) within total meningeal cells and in meningeal ILCs (Supplemental Figure 1). Metformin also expanded the pool of ILC2 (*P* < 0.01 versus placebo treatment; *P* < 0.0001 versus sham), while inhibiting both ILC1 (*P* < 0.01 versus placebo treatment; not significantly different from sham) and ILC3 (*P* < 0.05 versus placebo treatment; not significantly different from sham) after TBI (Figure 5, A and B). Further delineation of the meningeal ILC2 population after TBI revealed a distinct increase in GATA3^+^Lin^–^IL-10^+^ ILC2 following metformin treatment (*P* < 0.001 versus placebo treatment; *P* < 0.01 versus sham) (Figure 5C).

The increased total number of ILCs and ILCs as a percentage of total leukocytes were associated with elevated expression of the proliferation marker Ki-67 (Supplemental Figure 3, A and B) and the cellular adhesion molecule ICAM-1 (Supplemental Figure 3, C and D). Of note, postinjury administration of metformin increased the percentage of Ki67^+^ ILC2 (*P* < 0.0001 versus placebo-treated TBI; *P* < 0.001 versus sham), while simultaneously suppressing Ki67^+^ ILC1 (*P* < 0.0001 versus placebo-treated TBI; *P* < 0.0001 versus sham) and Ki67^+^ ILC3 (*P* < 0.0001 versus placebo-treated TBI; *P* < 0.0001 versus sham) (Supplemental Figure 3B). Metformin also increased the percentage of ICAM-1^+^ ILC2 (*P* < 0.0001 versus placebo-treated TBI; not significantly different from sham) while reducing the percentage of ICAM-1^+^ ILC1 (*P* < 0.0001 versus placebo-treated TBI; *P* < 0.0001 versus sham) and ICAM-1^+^ ILC3 (*P* < 0.0001 versus placebo-treated TBI; *P* < 0.0001 versus sham) (Supplemental Figure 3D).

In parallel to changes in ILCs, placebo-treated mice exhibited an increased presence of Th1 (*P* < 0.0001 versus sham) and Th17 (*P* < 0.0001 versus sham) with a concomitant reduction in Treg (*P* < 0.05 versus sham) after TBI (Supplemental Figure 4). Intracisternal administration of metformin at 2 hours after TBI reduced the number of Th1 (*P* < 0.0001 versus placebo treated; not significantly different from sham) and Th17 (*P* < 0.0001 versus placebo treated; not significantly different from sham) cells, while the number of Treg in the pericontusional cortex was increased (*P* < 0.01 versus placebo treated; not significantly different from sham) (Supplemental Figure 4).

The differential regulation of immune responses by intracisternal, postinjury administration of metformin was associated with neurobehavioral improvements in motor and psychiatric function after TBI (Supplemental Figure 5). Specifically, metformin decreased the time to traverse a beam (*P* < 0.01 versus placebo treatment), reduced the number of foot steps (*P* < 0.05 versus placebo treatment), and attenuated foot slips (*P* < 0.05 versus placebo treatment) in the narrow-beam test. Metformin also enhanced the time (*P* < 0.05 versus placebo treatment), distance (*P* < 0.05 versus placebo treatment), and maximal revolutions per minute achieved (*P* < 0.05 versus placebo treatment) on the rotarod. Finally, mobility time was improved by metformin treatment in the tail-suspension test for depression (*P* < 0.05 versus placebo treatment).

## Discussion

TBI is a worldwide public health issue, with an incidence exceeding breast cancer, AIDS, multiple sclerosis, and spinal cord injury combined (1). Preventative measures reduce injury occurrence and/or severity, yet one-third of hospitalized TBI patients die from secondary pathological processes that develop during supervised medical care (25). Progressive neurodegeneration is a frequent long-term sequelae of TBI, with over 2% of the population coping with the neurological consequences of a prior injury, including a heightened risk of incident dementia and cognitive dysfunction (26–28). Inflammation, a highly conserved response to injury, temporally paralleled delayed white matter loss and poor functional outcomes for years after a single TBI in humans and for over 1 year after controlled cortical impact (CCI) in rodents (29–31); however, administration of nonselective antiinflammatory therapies (e.g., corticosteroids) failed to improve patient outcomes after neurotrauma (32). Thus, targeted therapeutic approaches are needed to limit the deleterious aspects and promote the beneficial aspects of immune activation after acute brain injury. In this report, we identify AMPK-dependent suppression of proinflammatory ILC1 and ILC3 expansion, with a concomitant increase in the expansion of IL-10 expressing regulatory ILC2, within the meninges and CSF after experimental or clinical neurotrauma.

The meninges abut CSF, providing CNS-resident ILCs a strategic anatomical location to serve as cerebral immune gatekeepers, relaying information from the brain to the immune system. In line with this assertion, increased CSF levels of high mobility group box protein 1 (HMGB1), a damage-associated molecular pattern molecule (DAMP) that we identified as predictive of poor outcomes in TBI patients (33, 34), enhanced colonic ILC3 to exacerbate colitis (35). Similarly, elevated CSF levels of IL-1β, a potent inducer of ILC3 activation, correlated with the development of cerebral edema and poor neurological outcomes after human TBI (36). Thus, cytokines and DAMPs originating within injured brain tissue may enter the CSF and activate ILCs to coordinate the timing and magnitude of peripheral immune cell entry into the brain.

The pattern of ILCs within dura and CSF derived from TBI patients was mirrored by an increased number of ILC1 and ILC3 within the murine meninges after experimental TBI, indicative of a conserved immune response between clinical and experimental neurotrauma. In line with the notion that ILC1 and ILC3 generate type I and type III proinflammatory responses, respectively, we showed classical type 1 (Th1) and type III (Th17) lymphocytes infiltrated the CNS within the first days after injury and persisted for weeks after experimental TBI (37). Notably, elevated expression of IL-17, the signature cytokine of Th17 cells, temporally mirrored ILC3 expansion after TBI (38), while loss of T-bet in NKp46^+^ ILCs impaired the CNS infiltration of myelin-reactive Th17 cells, reduced neuroinflammation, and attenuated white matter loss within the spinal cord using a murine model of multiple sclerosis (39). Thus, therapies that limit proinflammatory ILC1/ILC3 activation may provide an innovative strategy to limit the detrimental effects of neuroinflammation after TBI.

ILC2 may exhibit phenotypically and functionally distinct subtypes that elicit context-dependent functions (40). For example, immunosuppressive ILC2 may release IL-13 to stimulate the development of immunosuppressive myeloid–derived suppressor cells, whereas inflammatory ILC2 produce IL-5 to enhance the cytotoxic activity of eosinophils to suppress tumor progression (41–43). The IL-33 receptor ST2 is highly expressed by ILC2, while IL-33, an injury-inducible cytokine that promotes the activation and effector functions of ILC2, improves recovery following CNS injury (44, 45). Herein, we observed that posttreatment with exogenous IL-33 enhanced the number of ILC2 via an AMPK-dependent pathway after TBI. Importantly, the stimulatory effect of IL-33 on ILC2 and the suppressive effect on ILC1/ILC3 was lost in Rag1^–/–^ IL2rg^–/–^, but not Rag1^–/–^ mice, suggesting direct action at the level of ILCs rather than actions via intermediary cell types. Further analysis identified an AMPK-regulated ILC2 population that mirrors the identification of a molecularly distinct subtype of ILC2, deemed ILC2_10_ cells, which express classical ILC2 markers and the regulatory cytokine IL-10 in response to IL-33 treatment (46, 47). These cells also match the recently described IL-10 expressing regulatory ILC (ILC_reg_) that suppress activation of ILC1 and ILC3 in the context of intestinal inflammation (48).

Metformin, a well-tolerated, US Food and Drug Administration–approved (FDA-approved) drug that serves as a first-line treatment option in type II diabetic patient, was administered via an intracisternal route to preferentially target ILCs while minimizing peripheral effects. Peripheral administration restores AMPK activation in the brain (49–51), while oral administration of 150 mg/kg metformin is detectable within the CSF (36.6 μmol/L) and forebrain (4.9–7.7 nmol/g tissue) within 6 hours (49).These findings illustrate that metformin readily crosses the BBB, and they suggest potential translational value of AMPK activators after TBI. We showed that posttreatment with metformin restored AMPK activation within meningeal ILCs, enhanced regulatory ILC2, and suppressed proinflammatory ILC1/ILC3 to improve neurological outcomes after TBI. These ILC changes mirrored shifts in the phenotypic profile of infiltrated T cells, with elevations in Th1 and Th17 cells observed within the pericontusional cortex after TBI. Conversely, intracisternal delivery of metformin suppressed proinflammatory T cells and enhanced Treg production, consistent with the generation of regulatory ILC2. Thus, immunometabolic reprogramming via AMPK activation may provide a clinically amenable therapeutic strategy to direct the selective expansion of regulatory ILC2, which in turn may enhance immune resolution.

Increased expression of Ki-67, a cellular proliferation marker, and ICAM-1, an adhesion molecule critical for ILC recruitment, were elevated within ILC1/ILC3 after experimental TBI. Coupled with an increased number of total meningeal ILCs, ILCs may locally proliferate and/or migrate into the meninges from the periphery after TBI. An elevated presence of ILCs also was observed within CSF of TBI patients, although we cannot distinguish whether these cells represent resident ILCs exiting the meninges or infiltrating cells entering the CNS. Interestingly, metformin elevated both Ki-67 and ICAM-1 expression specifically within ILC2 and normalized expression of these markers within ILC1/ILC3 after TBI, suggesting a possible role for AMPK in the regulation of ILC proliferation and/or migration after TBI. In addition, ILCs are highly plastic (52), providing another potential mechanism to explain changes in ILCs after TBI. Both human and mouse ILC3 can reversibly differentiate toward ILC1, while human ILC2 reversibly convert into ILC1 during type I inflammatory conditions (11, 53–56). Moreover, ILC2 may display an ILC3 phenotype under inflammatory conditions in mice (40). The interchangeable conversions of ILCs are dependent on the release of proinflammatory (e.g., IL-12, IL-1β) and antiinflammatory (e.g., IL-4) cytokines that may be regulated by AMPK, providing unexplored mechanism of ILC regulation.

A number of caveats warrant further discussion. Dura obtained from TBI patients provides an unmatched opportunity to directly assess the potential translational value of our observations in the murine CCI model; however, an insurmountable limitation of our clinical studies is the lack of nontraumatic control dura for comparison. Nonetheless, the pattern of ILCs observed in human dura after TBI mirrored our preclinical findings, supporting the validity of our preclinical model. The source of CSF is another consideration as trauma CSF was collected from an extraventricular drain, whereas control CSF from NPH patients were obtained by lumbar puncture. Although we analyzed the identical volume, CSF likely circulates slower in the lumbar space, as compared with the ventricles, which could potentially lead to underestimation of ILCs after lumbar puncture. The role of age on ILCs remains another potential caveat, as NPH patients were significantly older than the TBI population in our cohort (60.7 years versus 26.3 years, *P* < 0.001). In our preclinical studies, we directly administered drugs into the CSF in an attempt to circumvent peripheral effects (e.g., glycemic regulation by metformin) and IL-33–stimulated AMPK activation in ILCs; however, we cannot definitely conclude that behavioral improvements after metformin treatment were entirely dependent on local effects at the level of ILCs. Indeed, repeated, i.p. administration of metformin enhanced long-term spatial memory after experimental TBI (57). Thus, it is possible that the observed changes in ILCs may be, at least in part, secondary to direct neuroprotective effects and/or direct effects on infiltrating lymphocytes. Future work will further delineate the cellular mechanisms underlying the neuroprotective effects of metformin.

In conclusion, our study identifies meningeal ILCs as cellular sentinels within the CNS, relaying perturbations within the CNS to generate AMPK-dependent, context-specific immunity after TBI. Beyond the establishment of a conceptual framework to potentially explain the timing, specificity, and magnitude of cerebral immunity within an otherwise immune privileged CNS, our studies possess high translational potential. Given the association between chronic immune activation and poor TBI outcomes (3), ILCs may provide CSF-derived, acute phase prognostic biomarkers to prospectively diagnose patients at increased risk of developing progressive neurological injury. Defining the temporal pattern and functional ramifications of ILC activation also may identify a pharmacodynamic biomarker to monitor the efficacy and biological function of a given therapy. Toward this end, our studies support the clinical repurposing of established AMPK-activating drugs as immunotargeted therapeutics after neurological injury. While metformin is clinically approved and possesses immediate translational value, it is notable that the glucose-lowering effects of metformin may be independent of AMPK (58). Thus, the development of more selective AMPK activators may selectively induce counter-inflammatory ILC2 expansion without the associated risks of hypoglycemia in critically ill patients (59).

## Methods

### Experimental design.

Mice were used as the primary research subject in controlled laboratory experiments. CSF and dura from moderate-to-severe neurotrauma patients were used to extend the translational relevance of preclinical modeling studies. We utilized a double-blinded study design whereby mice were assigned a unique subject number and then randomized in a predetermined manner by a blinded study coordinator. Power analyses were conducted a priori to determine sample sizes using α = 0.05 and β = 0.10, using quantification of meningeal ILC subtypes or behavioral outcomes as the primary endpoints in preclinical studies. ILC subtypes were used as the primary endpoint in clinical studies. Blinded investigators performed all data acquisition of outcome measures. Following final data acquisition, mice were decoded and final analyses were performed. No experimental subjects were removed from the study, and all data were included in the final analysis.

### Animal model.

Adult male C57Bl/6J (The Jackson Laboratory, stock no. 000664), NOD/ShiLtJ (The Jackson Laboratory, stock no. 001976), AMPKα1^–/–^ (derived from founders provided by Benoit Viollet, L’Institut Chochin, Paris, France), Rag1^–/–^ (The Jackson Laboratory, stock no. 003729), or Rag1^–/–^IL2rg^–/–^ (The Jackson Laboratory, stock no. 007799) mice were subjected to a sham injury or CCI, as detailed by our laboratory (37). Briefly, mice were anesthetized using 3% isoflurane and maintained with 1.5%–2% isoflurane throughout all surgical procedures. Anesthetized mice were placed in a stereotaxic frame, and a craniotomy was made in the right parietal bone midway between the bregma and lambda with the medial edge 1 mm lateral to the midline, leaving the dura intact. Mice were impacted at 3 m/s with a 85 ms dwell time and 3.0 mm depression using a 3 mm diameter convex tip (PinPoint PCI3000 Precision Cortical Impactor, Hatteras Instruments). Sham-operated mice underwent the identical surgical procedures but were not impacted. The skin incision was closed, and mice were allowed to recover in a clean, warm cage. Body temperature was maintained at 37°C using a small-animal temperature controller throughout all procedures (Kopf Instruments). Food and water were provided ad libitum.

### Collection of patient specimens.

CSF was obtained from 6 consecutive severe adult TBI patients (Glasgow coma scale 3t-8) receiving extraventricular drainage due to elevated intracranial pressure within the Department of Neurosurgery at the Medical College of Georgia (Supplemental Table 1). Control CSF was obtained from NPH patients via lumbar puncture. A 1 cm^2^ dura section was surgically excised from patients requiring a hemicraniectomy due to a TBI. Demographic data is provided in Supplemental Table 2. Dura samples were placed in sterile saline within the operating room and immediately transported to the laboratory on ice. Patient specimens were collected from adults without regard to age, race, sex, or socioeconomic status. Samples were immediately placed on ice and transported to the laboratory for ILC analyses.

### Isolation of the cerebral meninges.

Meninges were isolated following sham or TBI, as previously described (60). Briefly, mice were anesthetized with 5% isoflurane and transcardially perfused with ice-cold saline. The skin and muscles were removed, and the skull was carefully cut using angled scissors, avoiding damage to underlying brain tissue. Both brain and the removed bone flap were placed in a petri dish containing DMEM on ice. Meninges were peeled from the brain surface and inside of the skull in a medial to lateral orientation. Next, the brain was divided into the cerebellum, brain stem, and cerebrum, and meninges were collected from the cerebral ventricles, brain stem, and cerebellar folds. Collected tissue was dissociated with the aid of a wide-tip plastic plunger and filtered through a 100 μm nylon cell strainer prior to further analysis.

### Intracisternal drug administration.

Placebo (PBS), 1 μg recombinant IL-33 carrier free (catalog 3626-ML/CF; R&D Systems), or 3 μg metformin (1,1-dimethylbiguanide hydrochloride, 97% purity; Sigma-Aldrich) in 5 μL PBS was injected into a subarachnoid space at a depth of 2 mm through a soft point located 3.5 mm rostral to bregma (coordinates AP = +3.5 mm, ML = 0 mm, and DV = 2.0 mm), as described (61). Drugs were administered at 2 hours after sham/TBI.

### Preparative and analytical flow cytometry.

For flow cytometry analysis, tissues were placed in a tissue culture dish with 1 mL PBS + 2% FCS, 2 mg/mL of collagenase type II, and 1 mg/mL of DNase type I for 30 minutes at 37°C. Samples were then sieved through a cell strainer (BD Biosciences), followed by centrifugation (252*g*, 5 minutes, 4°C) to prepare single-cell suspensions. Preparative cell sorts were performed on cells stained with fluorochrome-conjugated monoclonal antibodies using a Beckman Coulter MoFlo XDP cell sorter that can perform up to 7 color analysis at speeds of up to 30,000 cells/second and can collect from 1–4 separate cell populations. Sorted live cells were divided into 2 groups. The first group (1 × 10^4^ cells) were reserved for functional assays, as detailed in the following section. The remaining sorted cells were subjected to flow cytometry analysis using a LSRII 5-laser flow cytometer (Becton Dickinson). Briefly, cells were gated as Lin^−^CD45^+^ (mouse, catalog 103114, clone 30-F11; human, catalog 368532, clone 2D1) lymphocytes and a lineage cocktail of antibodies (all antibodies from BioLegend, unless otherwise noted) included FITC-conjugated anti-CD3 (mouse, catalog 100204, clone 17A2; human, catalog 300328, clone HIT3a), anti-CD4 (mouse, catalog 100406, clone GK1.5; human, catalog 300538, clone RPA-T4), anti-CD14 (mouse, catalog 123308, clone Sa14-2; human, catalog 367116, clone 63D3), anti-CD16 (mouse, catalog 101305, clone 93; human, catalog 302006, clone 3G8), anti-CD19 (mouse, catalog 152404, clone 1D3/CD19; human, catalog 302206, clone HIB19), anti-CD8 (mouse, catalog 140404, clone 53-5.8; human, catalog 344704, clone SK1), anti-CD15 (human/mouse, catalog 125611, clone MC-480), anti-CD20 (mouse, catalog 152108, clone SA271G2; human, catalog 302310, clone 2H7), anti-CD34 (human, catalog 343604, clone 561), and anti-CD203 (human, catalog 324622, clone NP4D6) were used for negative selection. Subsequently, ILC1 were identified as human (CD45^+^Lin^−^CD127^+^CD161^+^, Abcam, catalog ab197979) NKp44^+^ (BioLegend, catalog 325108, clone P44-8) mouse (Lin^−^CD127^+^IL-12Rβ2^+^ [mouse/human, R&D Systems, catalog FAB1959P-100, clone 305719]) cells, ILC2 were identified as human (CD45^+^Lin^−^CD127^+^GATA3^+^, BioLegend, catalog 653808, clone 16E10A23) CRTH2^+^ (Thermo Fisher Scientific, catalog PA5-20333) and mouse (Lin^−^CD127^+^GATA3^+^) cells, and ILC3 were identified as human (CD45^+^Lin^−^CD127^+^RORγt^+^AhR^+^) and mouse (Lin^−^CD127^+^RORγt^+^; mouse/human, Thermo Fisher Scientific catalog 17-6988-82, clone AFKJS-9) cells (all antibodies from BioLegend). Activated AMPK was quantified in permeabilized cells using an antibody directed against phosphorylated AMPK_α1/2_^Thr183/Thr172^ (Bioss, catalog bs-4002R-Cy7). For quantification of T cells, freshly harvested brain tissue was sieved through a 100 μM cell strainer and centrifuged (252*g*, 5 minutes, 4°C) to prepare single-cell suspensions. Cells were incubated with antibodies against the cell surface marker CD4. Following a PBS wash, cells were fixed and permeabilized using a Fixation/Permeabilization Concentrate (Affymatrix eBioscience) and then incubated with antibodies for intracellular labeling of IFN-γ, IL-4, IL-17, or FoxP3, as detailed by our laboratory (37). After a final wash, cells were analyzed by a LSRII 5-laser flow cytometer. Isotype-matched controls were analyzed to set the appropriate gates for each sample. For each marker, samples were analyzed in duplicate. To minimize false-positive events, the number of double-positive events detected with the isotype controls was subtracted from the number of double-positive cells stained with corresponding antibodies (not isotype control). Cells expressing a specific marker were reported as a percentage of the number of gated events. A population was considered positive for a specific marker if the population exceeded a 2% isotypic control threshold.

### Analysis of patient CSF.

CSF was immediately transported to the laboratory on ice, centrifuged (300*g*, 5 minutes, 4°C), and the supernatant was discarded. Cells were resuspended in 0.5% albumin in 2.0 mL of PBS, samples were centrifuged (300*g* for 5 minutes, 4°C), the supernatant as discarded, and the remaining cells were suspended in 300 μL of 0.5% albumin in PBS for analysis. We analyzed patient samples by volume with ~3 × 10^3^ cells/mL observed in NPH patients and approximately 6 × 10^5^ cells/mL in TBI patients. Human ILCs were enriched by removing lineage-positive cells (CD3^+^, CD5^+^, CD19^+^) using a MACS cell sorting system (Miltenyi Biotec). Sorted human ILCs were then divided into 2 portions. A small group of cells (1 × 10^4^ cells) was used for functional assays, and the remainder of cells were stained using a panel of fluorochrome-conjugated antibodies to detect ILCs, as noted in the previous section.

### Functional ex vivo assays.

Sorted live cells from human tissue were collected, as detailed in the previous sections. ILCs (CD45^+^Lin^–^CD127^+^) were plated in 96 microplates at 1 × 10^4^ cells per well. Cells were treated with various cytokines (including IL-15 [Thermo Fisher Scientific, catalog PMC0155], IL-12 [BioLegend, catalog 577004], IL-18 [Thermo Fisher Scientific, catalog PMC0184], IL-23 [BioLegend, catalog 589004], IL-1β [Thermo Fisher Scientific, catalog PMC0816], IL-33 [BioLegend, catalog 580504], IL-25 [BioLegend, catalog 587304], and TSLP [R&D Systems, catalog 555-TS-010/CF]) at a concentration of 20 ng/mL and in the presence of Golgi Plug (BD Biosciences) for 24 hours. In addition, a nonstimulated control containing only medium was included. Finally, the functionality capacity to produce effector cytokines for ILC1s (IFN-γ/TNF-α) (anti–IFN-γ mouse, BioLegend, catalog 505810, clone XMG1.2; anti–IFN-γ human, BioLegend, catalog 502512, clone 4S.B3; anti–TNF-α mouse, BioLegend, catalog 506318, clone MP6-XT22; anti–TNF-α human, BioLegend, catalog 502920, clone Mab11), ILC2s (IL-5/IL-13) (anti–IL-5 mouse/human, Novus Biologicals, catalog NB100-2289AF700, clone TRFK5; anti–IL-13 mouse, Santa Cruz Biotechnology, catalog sc-393365, clone A-9; anti–IL-13 human, Santa Cruz Biotechnology, catalog sc-390676, clone F-6), and ILC3s (IL-17/IL-22) (anti–IL-17 mouse/human, Santa Cruz Biotechnology, catalog sc-374218, clone G-4; anti–IL-22 mouse, BioLegend, catalog 516411, clone Poly5164; anti–IL-22 human, BioLegend, catalog 366704, clone 2G12A41) was analyzed by flow cytometry.

### Neurobehavioral outcomes.

Motor coordination was assessed using an accelerating rotarod task, as described by our laboratory (62). Briefly, mice were trained for 3 consecutive days prior to injury and reassessed at day 3 after TBI. Mice were placed on an accelerating rotarod cylinder that was slowly increased from 4 to 40 rpm over a 5-minute trial. The length of time balance was maintained while walking on top of the drum was recorded. A trial ended once the mouse fell off or gripped the cylinder for 2 consecutive revolutions without walking. Motor coordination was further determined by measuring the time required to traverse a stationary 1 m narrow beam (6 mm width). Depressive behavior was assessed by mobility time in the tail-suspension test. Each mouse was tested 3 times, and the average time to traverse the beam was recorded. All behavioral analyses were done by blinded investigators.

### Statistics.

All data were analyzed using GraphPad Prism 8 software. Multigroup comparisons were made using a 1-way ANOVA with adjustments for multiple comparison. Data were further analyzed by Tukey’s post hoc test. Two-group comparisons were analyzed by 2-tailed Student’s *t* test. Results are expressed as mean ± SD. *P* < 0.05 was considered to be statistically significant.

### Study approvals.

The IACUC at Augusta University approved all animal studies, in compliance with NIH guidelines. The IRB at Augusta University approved all studies involving the collection of patient specimens.

## Author contributions

BB, KV, and KMD conceived the study, oversaw all aspects of the research, and drafted the manuscript. KV, MB, and KA performed TBI modeling, neurobehavioral testing, and tissue preparation. BB, HK, and AM performed flow cytometry experiments. AW, KN, SN, SF, AFP, FLV, and JRV collected patient CSF and/or contributed patient dura samples. OA, MNH, and DCH contributed to intellectual input, data analysis, and manuscript preparation. KMD, BB, and KV drafted the manuscript. All authors edited and approved the final version of the manuscript.

## Supplementary Material

Supplemental data

## Version 1 01/11/2021

Electronic publication
