# Supplementary material for: AMPK induces regulatory innate lymphoid cells after traumatic brain injury
Source: JCI Insight. 2021 Jan 11;6(1):e126766. doi: 10.1172/jci.insight.126766 (PMC7821592; doi:10.1172/jci.insight.126766)
Supplement: Supplemental data [file jciinsight-6-126766-s016.pdf]

Supplemental Data

SEX	AGE	DIAGNOSIS
M	72	Normal pressure hydrocephalus
F	47	Normal pressure hydrocephalus
F	70	Normal pressure hydrocephalus
M	66	Normal pressure hydrocephalus
M	60	Normal pressure hydrocephalus
M	58	Normal pressure hydrocephalus
M	19	TBI – Motor vehicle accident (GCS = 3)
M	19	TBI – Motor vehicle accident (GCS = 7)
M	36	TBI – Motor vehicle accident (GCS = 7T)
M	18	TBI – Motor vehicle accident (GCS = 8)
M	38	TBI – Non-penetrating, secondary to projectile (GCS = 3T)
F	28	TBI – Motor vehicle accident (GCS = 8)

Supplemental Table 1. Demographic data of NPH and TBI patients used in CSF collection. Cerebrospinal fluid (CSF) was collected by lumbar puncture from six consecutive NPH patients while CSF was collected from six consecutive severe TBI patients requiring extraventricular drainage. Sex, age, cause of trauma, and Glasgow Coma Scale (GCS) score of patients at the time of sample collection are provided.

SEX	AGE	CAUSE OF TBI
M	47	Penetrating brain injury
M	21	Motor vehicle accident
M	unknown	Non-penetrating, secondary to projectile
F	56	Motor vehicle accident
M	44	Non-penetrating, secondary to projectile
M	28	Motor vehicle accident

Supplemental Table 2. Demographic data of TBI patients used in dura collection. Dura was surgically excised from six consecutive severe TBI patients requiring a hemicraniectomy due to TBI. Sex, age, and cause of trauma for patients are provided.

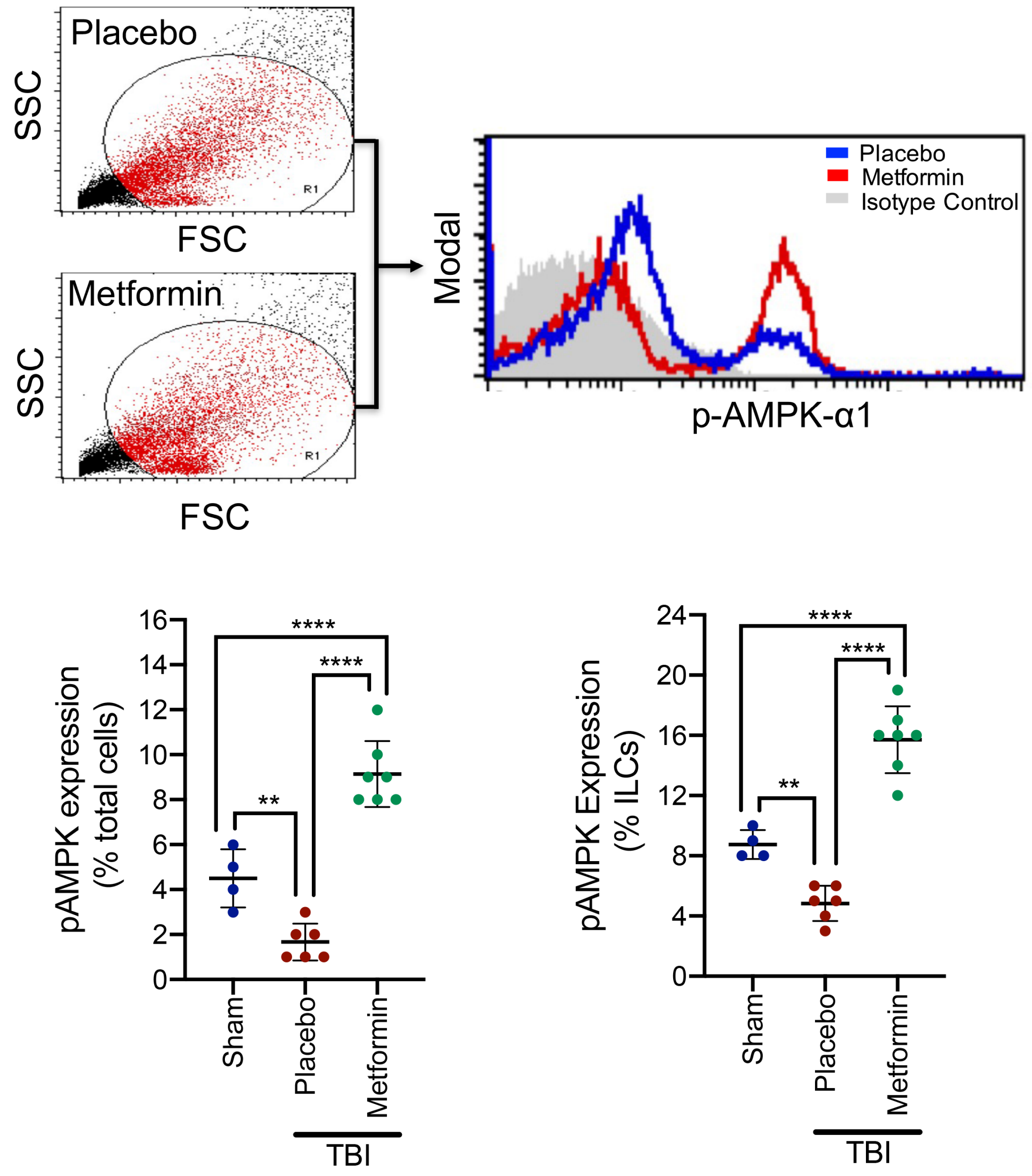

Supplemental Data Figure 1. Intracisternal administration of metformin increased meningeal AMPK activation after TBI. Placebo (PBS) or metformin (3 µg) was intracisternally administered to mixed sex C57Bl/6J mice at 2h post-TBI. Meningeal tissue was collected at day 5 post-TBI and phosphorylated AMPKα1 (p-AMPKα1) was quantified by flow cytometry. Grey shaded areas indicate isotype controls. Graphs indicate the % of total meningeal cells and % total ILCs that are positive for p-AMPKα1. Quantified data (n=4-6/group) are expressed as mean ± SD and were compared using a Student's t-test (**p<0.01, ****p<0.0001).

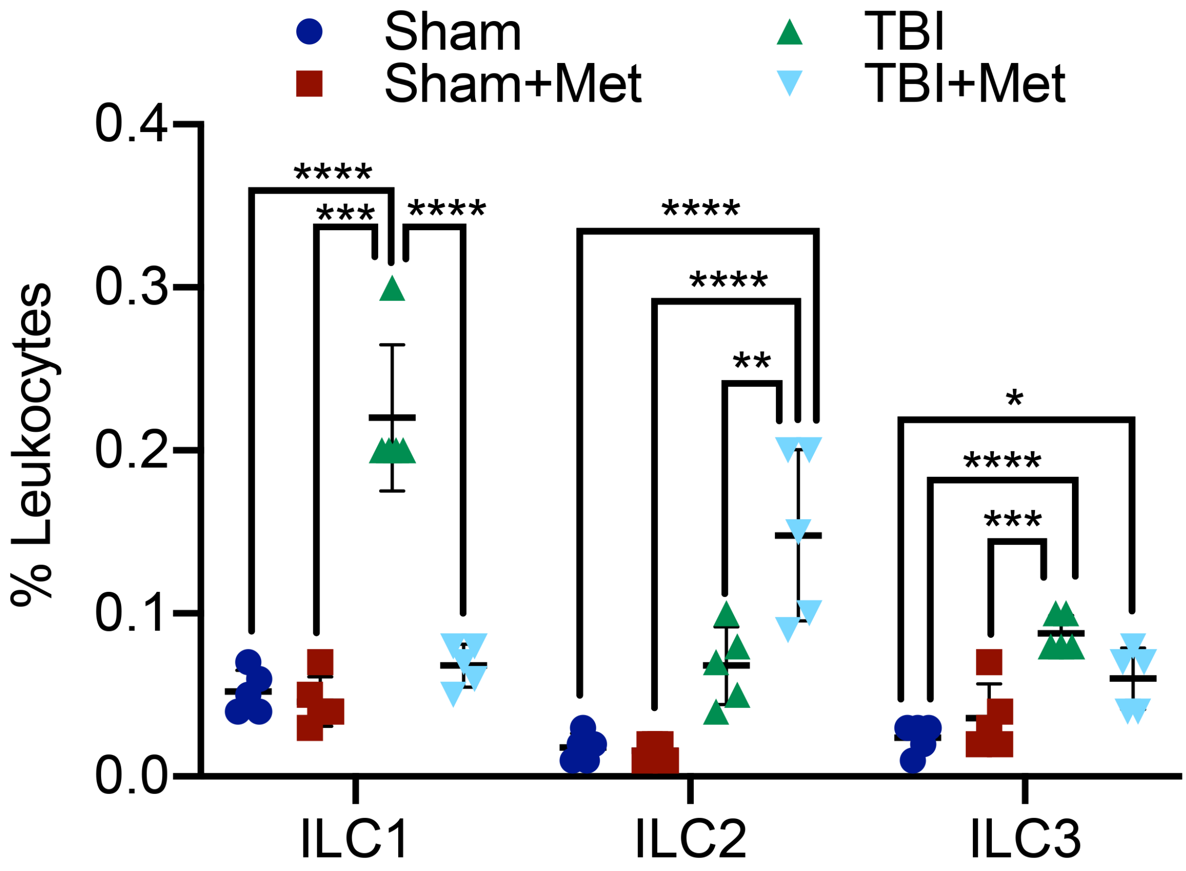

Supplemental Data Figure 2. Metformin increases CNS-resident ILC2 after experimental TBI. Placebo (PBS) or Metformin (3 µg) was intracisternally administered to mixed sex C57Bl/6J mice at 2h post-sham/TBI. Isolated meninges were analyzed by flow cytometry, as shown in Fig 3, at day 7 post-sham/TBI. Quantified data (n=5 mice/group), which depict ILC subtypes as % leukocytes, are presented as mean ± SD and compared using a One-Way ANOVA followed by Tukey's post-hoc test (**p<0.01, ****p<0.0001).

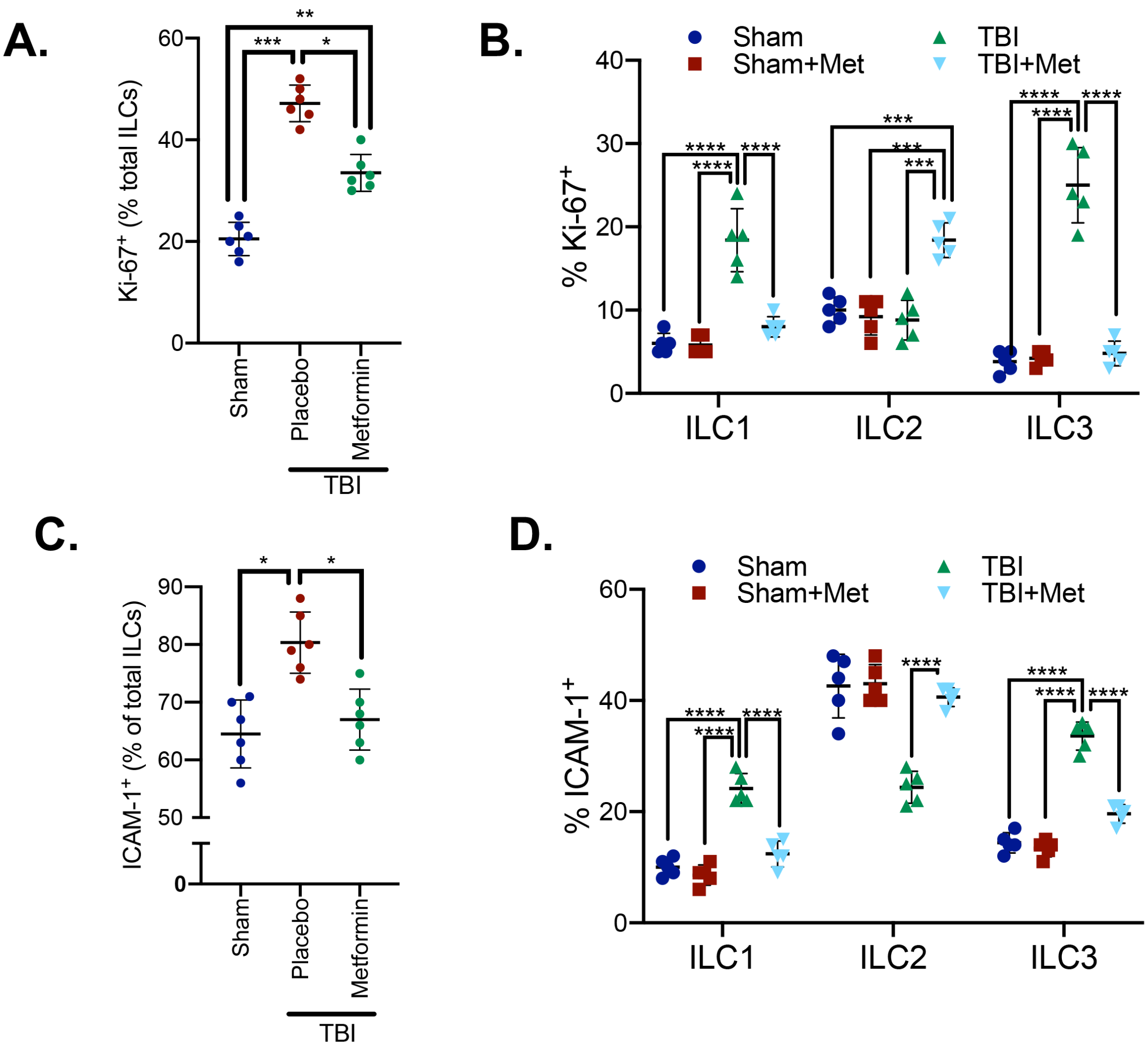

Supplemental Data Figure 3. Metformin suppresses ILC proliferation and migration. Placebo (PBS) or metformin (3 µg) was intracisternally administered at 2h post-TBI in mixed sex C57Bl/6J mice. At day 3 post-sham/TBI, ILCs were identified as $CD45^+$, lineage negative (Lin^-), $CD127^+$ lymphoid cells. Cellular proliferation in (**A., C.**) total ILCs and (**B., D.**) ILC subtypes was quantified using Ki-67 while migration was assessed using ICAM-1. Quantified data (n=5 mice/group) are shown in scatterplots. Data were compared using a One-Way ANOVA followed by Tukey's post-hoc test with corrections for multiple comparisons (*p<0.05, **p<0.01, ***p<0.001).

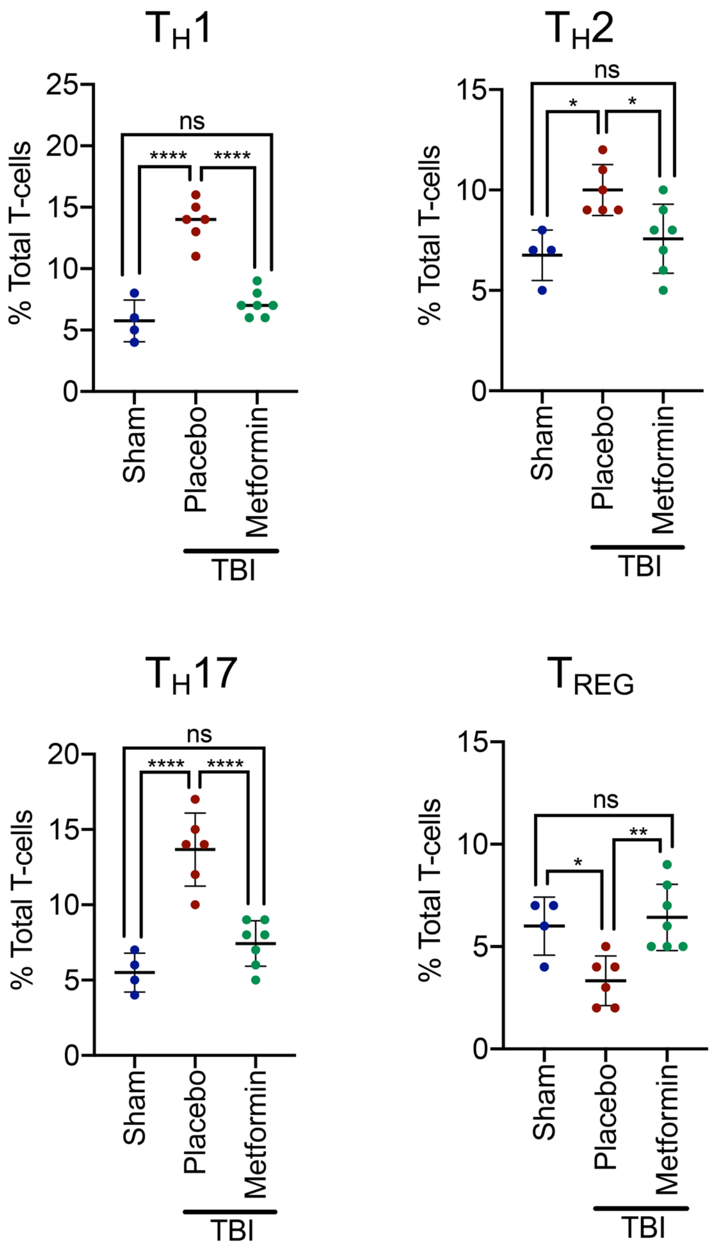

Supplemental Data Figure 4. Intracisternal administration of metformin modulates T-cell polarization within the brain after TBI. Placebo (PBS) or metformin (3 μg) was intracisternally administered at 2h post-TBI in mixed sex C57Bl/6J mice. At day 3 post-TBI, peri-contusional brain tissue was collected and T-cell polarization was assessed by flow cytometry. Scatterplots show quantified data for T_H1 ($CD4^+IFN\gamma^+$), T_H2 ($CD4^+IL\text{-}4^+$), T_H17 ($CD4^+IL\text{-}17^+$) and T_{REG} ($CD4^+Foxp3^+$). Data (n=4-7 mice/group) were compared using a One-Way ANOVA followed by Tukey's post-hoc test with corrections for multiple comparisons (*p<0.05, **p<0.01, ****p<0.0001).

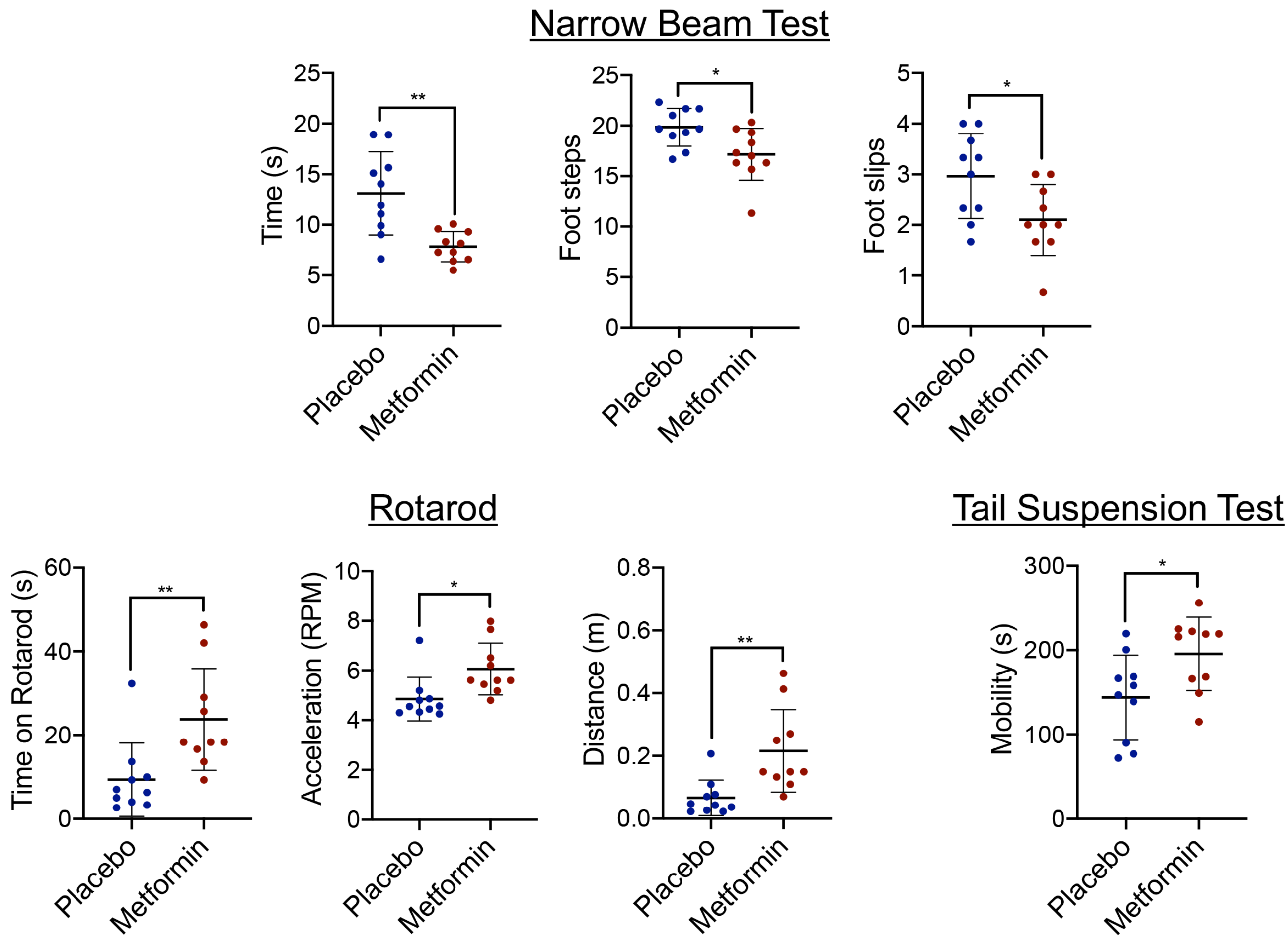

Supplemental Data Figure 5. Metformin improved neurological outcomes after TBI. Placebo (PBS) or metformin (3 μg) was intracisternally administered at 2h post-TBI. At day 3 post-TBI, motor function was assessed using the narrow beam test and the rotarod test. On day 4 post-TBI, depressive behavior was quantified using the tail suspension test. Scatterplots depict individual values (n=10/group) and mean ± SEM from two independent experiments. Statistical significance was determined using a Student's t-test (*p<0.05, **p<0.01).
